# The curse of angiopoietin-2 in ARDS: on stranger TI(E)des

**DOI:** 10.1186/s13054-018-1978-0

**Published:** 2018-02-25

**Authors:** Philipp Kümpers, Alexander Lukasz

**Affiliations:** Department of Medicine D, Division of General Internal Medicine, Nephrology, and Rheumatology, University Hospital Münster, Albert-Schweitzer-Campus 1, 48149 Münster, Germany

**Keywords:** Permeability, Lung, Pneumonia, ARDS, Endothelium, Angiopoietin-2, Tie2, Endothelial glycocalyx

## Abstract

Pulmonary inflammation and vascular leakage are hallmarks of acute respiratory distress syndrome (ARDS), a life-threatening condition, for which there is no specific pharmacologic treatment.

Recent literature suggests that leaky vessels in pulmonary infection and ARDS may be mediated through dysregulation of a non-redundant endothelial control pathway, the Tie2 receptor and its ligands, the angiopoietins.

This Viewpoint summarizes results from cell-based experiments, animal models and clinical studies underlining the potential of Tie2 targeted interventions in reducing infection-mediated pulmonary hyperpermeability.

Jack Sparrow, Captain of the Black Pearl and legendary pirate of the Seven Seas, must have been aware that only a watertight pirate vessel could outrun the Flying Dutchman as well as the heavily armed armada of the British Royal Navy and East India Trading Company.^1^ Thus, his crew needed significant knowledge and practice in caulking, a traditional technique whereby the gaps between the shell strakes or deck planks must be sealed using fibers, cord and pitch. Unlike early mariners, today’s physicians are probably less aware of leaky vessels—which may be because of the fact that they must not fear immediate shipwreck on the high sea—and, more importantly, they lack sealant.

## Vascular leakage in acute respiratory distress syndrome (ARDS)

Pulmonary inflammation and vascular leakage are hallmarks of ARDS, a life-threatening condition for which there is no specific pharmacologic treatment. ARDS may be triggered by a plethora of insults (direct or indirect) to the lungs. The insult results in increased epithelial (and particularly endothelial) permeability, which leads to “alveolar flooding” with a protein-rich edema fluid. The latter appears as otherwise unexplained bilateral radiographic opacities, a central component of the Berlin definition of ARDS. The resulting loss of gas exchange often requires ventilatory and critical care support, and remains a significant disease burden, with some estimates showing 200,000 cases each year in the USA with a mortality approaching 50% [1]. There are currently no disease-modifying therapies available, and the most effective advances in caring for ARDS patients have been in constant improvement to ventilator strategies [2].

In search of a molecular sealant, we have summarized recent literature showing that leaky vessels in pulmonary infection and ARDS may be mediated through dysregulation of a signaling axis based on angiopoietin (Angpt) and Tie2.

## Angpt/Tie2 ligand–receptor system

The vascular-associated receptor tyrosine kinase Tie2 and its agonist ligand Angpt-1 were discovered in the mid-1990s [3, 4]. Early studies in Angpt-1^−/−^ and Tie2^−/−^ knockout mice (which die *in utero* owing to severe vascular defects) revealed the importance of operational Angpt-1–Tie2 signaling for developmental angiogenesis [3, 4]. However, Angpt-1 was identified subsequently as a transdominant anti-permeability factor that protects the vasculature of adult mice from the plasma leakage induced by vascular endothelial growth factor and other inflammatory stimuli [5]. In contrast, Angpt-2 release from endothelial Weibel–Palade bodies disrupts constitutive Angpt-1–Tie2 signaling by preventing Angpt-1 from binding to the receptor [6–8], thereby promoting inflammation and permeability (see [9, 10] for detailed information).

## Angpt-2 level in plasma as a biomarker in ARDS

Our research team and other scholars have used commercial and customized immunoassays to investigate the plasma level of Angpt-2 as a vascular-specific biomarker in ARDS (and especially sepsis) for more than one decade [11, 12].

Van der Heijden et al. [13] were the first to describe that plasma levels of Angpt-2 were proportional to ARDS severity (lung injury score, pulmonary leak index, P_a_O_2_/F_i_O_2_ ratios) and correlated with the duration of mechanical ventilation. Calfee et al. [14] measured Angpt-2 levels in retained samples from 931 patients participating in the ARDS Network’s FACTT trial of a fluid-liberal *vs*. fluid-conservative management strategy in acute lung injury (ALI). In this large multicenter cohort of patients with ALI attributable to various causes, rising Angpt-2 levels over time were strongly and independently predictive of poor outcomes. However, subgroup analyses uncovered higher baseline levels of Angpt-2 in infection-related ALI (pneumonia and sepsis) compared with those in non-infected ALI. This finding suggests that there may be a considerable amount of circulating Angpt-2 in indirect ARDS—due to huge amounts of Angpt-2 arising from sustained systemic vascular inflammation (e.g., in sepsis)—compared with direct ARDS, in which moderate local release of Angpt-2 is accompanied by prominent epithelial injury. Agrawal et al. [15] showed that increased plasma levels of Angpt-2, measured upon admission to the emergency room or intensive care unit (ICU), predicted the development of ARDS accurately a median of 22 h before its onset in a mixed cohort of critically ill patients with prevailing infection (pneumonia or sepsis). Ong et al. found that the ratio of Angpt-2/Angpt-1, when added to established clinical and physiologic variables, significantly improved risk prediction in ALI patients [16]. In summary, the studies mentioned above suggest a strong association between increased or increasing Angpt-2 levels and mortality in ARDS. However, the value of Angpt-2 as a diagnostic marker for ARDS development has not been elucidated fully.

## Molecular control of the endothelial cell and its glycocalyx by Tie2

An increasing number of translational studies have shown consistently that maintenance of operational Angpt-1–Tie2 signaling counteracts (and withdrawal of Tie2 phosphorylation by Angpt-2 promotes) hyper-permeability through multiple-level effects on intracellular signaling, the cytoskeleton, and junction-related molecules, culminating in the formation of intercellular gaps between endothelial cells [17] (see [9, 10] for detailed information). Recently, we showed that Angpt-2 also mediates breakdown of the endothelial glycocalyx, a protective carbohydrate-rich, gel-like mesh of large anionic polymers that lines the luminal side of the endothelium along the entire vascular tree. Mechanistically, Angpt-2 causes heparanase secretion from distinctive cellular storage pools with consecutive enzymatic degradation of the glycocalyx [18]. A groundbreaking translational study by Schmidt et al. provided compelling evidence that prevention of heparanase-mediated degradation of the pulmonary endothelial glycocalyx is sufficient to eliminate vascular hyperpermeability and leukocyte adhesion in murine endotoxemia [19]. Han et al. were the first to show a protective effect of Tie2-targeted therapy on glycocalyx integrity in a clinically relevant peritonitis model in mice. Using a novel humanized monoclonal immunoglobulin G1 (IgG1) Angpt-2-binding antibody (termed ABTAA), they blocked the upregulation of heparanase and subsequent glycocalyx damage, which was paralleled by attenuated ALI and improved survival. Unlike other conventional Angpt-2 antibodies, ABTAA not only scavenges Angpt-2, but also induces Tie2 phosphorylation and translocation to cell-cell contact sites (just like Angpt-1) [20]. Those studies and our investigations using recombinant Angpt-1 [18] or the synthetic Tie2-peptidomimetic Vasculotide [21] suggest that exogenous activation of Tie2 (which we and others [20, 22] consider superior to sole inhibition of Angpt-2) may serve as a potential molecular sealant for leaky vessels.

## Tie2-targeted therapy as sealant for leaky vessels

The interesting question is whether a leaky vessel can be mended at stormy sea or if dry dock-like conditions are required (i.e., whether a Tie2-activating drug must be administered before the vascular damage is too grave to repair). Unfortunately, Tie2 expression declines rapidly in wide-ranging models of leak-associated infections, including anthrax, influenza, malaria, and sepsis [23–25]. It is, therefore, important to investigate the protective effect of Tie2-targeted therapy administered *after* disease onset. This important aspect has been largely neglected in the literature. However, Vasculotide, a synthetic polyethylene glycol-clustered Tie2-binding peptide developed with the aim of tetramerically binding and clustering Tie2 receptors in an ‘Angpt-1-like’ manner, has been shown to reduce mortality if started 2 h after induction of abdominal sepsis or injection of endotoxin, respectively [21, 22]. The peptide (named T7) originally described by Tournaire R. et al. [26], was found to bind with high affinity outside of the shared Angpt1, Angpt2 ligand binding pocket and is thus not able to displace the endogenous ligands. In our hands, Vasculotide administration results in sustained activation of Tie2 for more than 36 h *in vivo* [21]. Likewise, ABTAA improved survival when started 6 h after induction of three models of abdominal sepsis [20]. Although this survival benefit seen with the two compounds is not necessarily and exclusively due to preservation of pulmonary permeability, improved survival was consistently associated with preserved pulmonary function and structure. However, an important finding is that the reduced abundance of Tie2 protein induced by endotoxemia or sepsis can be overcome by increased activation of the remaining Tie2 molecules if Vasculotide or ABTAA is given [20–22].

In a recent issue of *Critical Care*, Gutbier et al. (Berlin, Germany) set sail to explore the effects of Vasculotide in pneumococcal-induced ALI [27]. For proof of principle, they challenged lung endothelial cells and isolated perfused mouse lungs with pneumolysin, a toxin and putative virulence factor of *Streptococcus pneumoniae*. Pretreatment with Vasculotide abolished the loss of endothelial integrity and hyper-permeability evoked by pneumolysin *in vitro* and *ex vivo*. Although not shown by the authors, it is likely that the pneumolysin-induced hyperpermeability is (at least in part) mediated by Angpt-2. Previous *in vitro* experiments have shown that human microvascular pulmonary endothelial cells release Angpt-2 rapidly if challenged with the major components of cell walls of Gram-positive (pneumonia-associated) bacteria such as lipoteichoic acid and peptidoglycans [28].

Next, Gutbier et al. infected mice transnasally with *S. pneumoniae* and injected Vasculotide or vehicle repeatedly when pneumonia was established (starting point was 22 h after *S. pneumoniae* inoculation). In this clinically meaningful model and dosing regimen, Vasculotide reduced transvascular albumin leakage and edema formation in the lungs dose-dependently 24 h and 48 h post-infection. The novelty and importance of this finding is that Vasculotide had sealed the leaky vasculature already 2 h after its administration. Whether this protective effect of Vasculotide translates into survival benefit in pneumococcal pneumonia (not done), as has been impressively shown in a murine model of severe influenza infection, will be interesting [29]. Here, the authors showed that Vasculotide, even if administered as late as 72 h after infection, reduced lung edema, arterial hypoxemia and the apoptosis of endothelial cells in the lung. Interestingly, post-treatment with Vasculotide did not alter the pulmonary recruitment of immune cells, cytokine release or the systemic immune response in the two aforementioned studies. This finding corroborates previous results in an endotoxemia-induced ALI model, suggesting that vascular leak and transmigration of immune cells may be regulated independently, and/or that leukocyte recruitment was accomplished before Tie2-targeted therapy was initiated [22, 29]. The effect of Tie2 activation on inflammation may also depend on the animal/disease model, as pre-and post-treatment with ABTAA blunted the cytokine storm in polymicrobial abdominal sepsis (CLP model) but not in endotoxemia [20]. Further studies are needed to address this important issue in more detail.

## Perspectives for further research

Most therapeutic studies in animals have focused on sepsis (i.e., indirect ARDS). Accordingly, little is known about the Tie2-dependent interaction between endothelial and epithelial cells in direct ARDS. Syed et al. reported that Tie2 and Angpt-1 is expressed by murine type-2 alveolar epithelial cells *in vitro* [30]. Another research team showed that tumor necrosis factor (TNF)-α treatment increased Angpt-2 expression, and that knockdown of Angpt-2 ameliorated TNF-α-induced apoptosis in human alveolar epithelial cells *in vitro* [31]. Therefore, the lung may be uniquely dependent on Tie2 signaling to maintain endothelial and epithelial integrity.

David et al. showed, in murine endotoxemia and sepsis, that lung-specific downregulation of Angpt-2 by small interfering RNA (siRNA) attenuated injury to distant organs, including the kidney. Intriguingly, renal function was improved although the siRNA delivery did not affect renal expression of Angpt-2 [32]. This finding suggests that pulmonary Angpt-2 release may enhance remote-organ damage in ARDS. Further studies are needed to better understand the role of the Angpt/Tie2 system in crosstalk at cellular and organ levels.

## Conclusion and outlook

Accumulating evidence from basic-science and animal studies suggest that Tie2-activating drugs could have great potential to seal leaky vessels in human disease syndromes such as sepsis and ARDS. However, critical illness is often described as a “graveyard” for pharmaceutical trials, in part because markers of molecular disease, as in “tailored” cancer therapy, are lacking or not readily available in the ICU. Here, detection of high plasma levels of Angpt-2, maybe as a point-of-care test, may offer a relatively straightforward way to recruit a reasonably homogenous group of critically ill patients with deactivated Tie2 signaling. Vasculotide and ABTAA are probably the most promising candidate compounds for this indication, although the latter has higher production costs and a higher immunologic risk profile. Specific side effects have not been reported so far. Therefore, we believe that Tie2-targeted therapies have the potential to lift the curse of Angpt-2 from ARDS. The ultimate proof of these concepts will require carefully designed clinical trials. So, let’s set sail...on stranger Ti(e)des.

## Abbreviations

ABTAA: Angpt2-binding and Tie2-activating antibody
ALI: Acute lung injury
Angpt: Angiopoietin
ARDS: Acute respiratory distress syndrome
FACCT: Fluid and catheters treatment trial in ARDS
ICU: Intensive care unit
TNF-α: Tumor necrosis factor-α

## Footnotes

1 *Pirates of the Caribbean: on stranger tides* is a fantasy swashbuckler film produced by Walt Disney Pictures and released in the USA on 20 May 2011.
